# Supplementary figures and images for: Identification of candidate long non-coding RNAs and mRNAs associated with heart aging in mice
Source: PeerJ. 2025 Dec 2;13:e20433. doi: 10.7717/peerj.20433 (PMC12679924; doi:10.7717/peerj.20433)

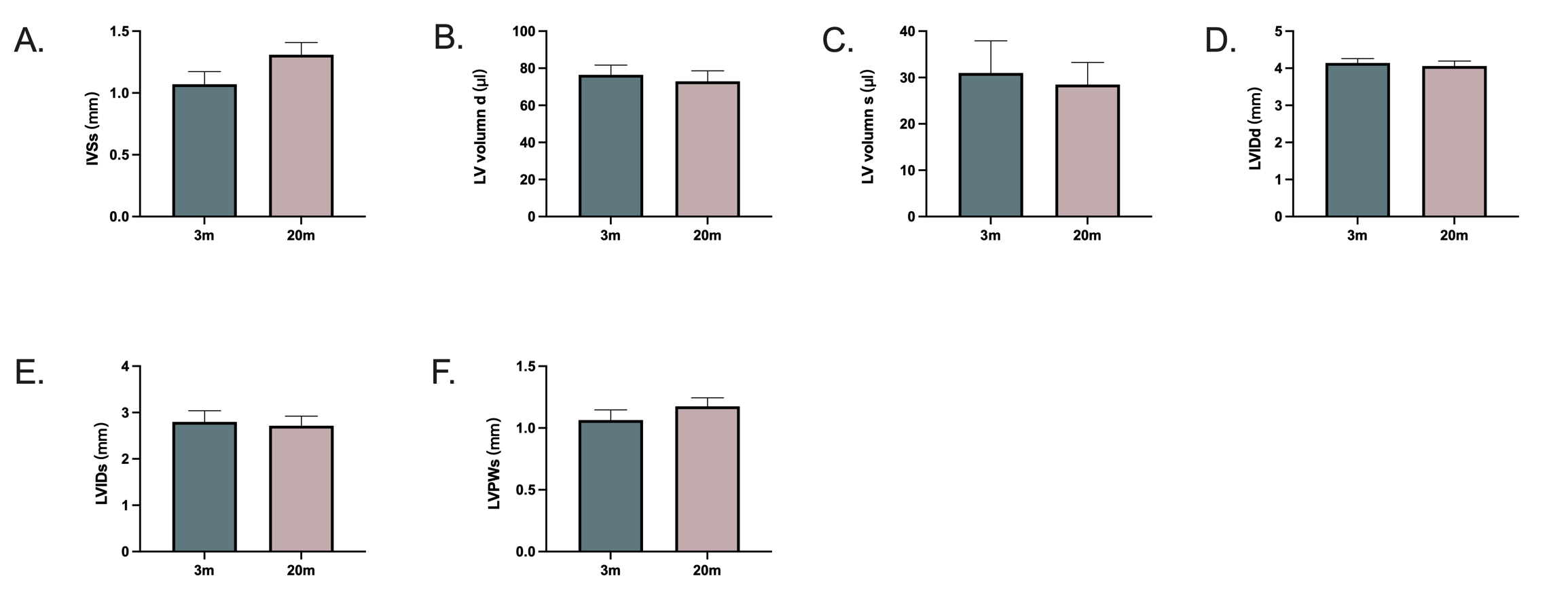

Supplement: Supplemental Information 1 [file peerj-13-20433-s001.png]
